# Low Ejection Fraction Predisposes to Contrast-Induced Nephropathy after the Second Step of Staged Coronary Revascularization for Acute Myocardial Infarction: A Retrospective Observational Study

**DOI:** 10.3390/jcm9061812

**Published:** 2020-06-10

**Authors:** Michał Chyrchel, Przemysław Hałubiec, Agnieszka Łazarczyk, Olgerd Duchnevič, Michał Okarski, Monika Gębska, Andrzej Surdacki

**Affiliations:** 1 Second Department of Cardiology, Jagiellonian University Medical College, 2 Jakubowskiego Street, 30-688 Cracow, Poland; mchyrchel@gmail.com; 2 Students’ Scientific Group at the Second Department of Cardiology, Jagiellonian University Medical College, 2 Jakubowskiego Street, 30-688 Cracow, Poland; przemyslawhalubiec@gmail.com (P.H.); agnieszka013@neostrada.pl (A.Ł.); olgierd.du@gmail.com (O.D.); julian98.mo@gmail.com (M.O.); 3 Department of Cardiology, District Hospital, 36 Jagiellońska Street, 97-500 Radomsko, Poland; gebska.monika@gmail.com

**Keywords:** contrast-induced nephropathy, acute myocardial infarction, staged coronary revascularization, ejection fraction

## Abstract

Patients who develop contrast-induced nephropathy (CIN) are at an increased short-term and long-term risk of adverse cardiovascular (CV) events. Our aim was to search for patient characteristics associated with changes in serum creatinine and CIN incidence after each step of two-stage coronary revascularization in patients with acute myocardial infarction (AMI) and multivessel coronary artery disease undergoing staged coronary angioplasty during hospitalization for AMI. We retrospectively analyzed medical records of 138 patients with acute myocardial infarction without hemodynamic instability, in whom two-stage coronary angioplasty was performed during the initial hospital stay. In-hospital serum creatinine levels were recorded before the 1st intervention (at admission), within 72 h after the 1st intervention (before the 2nd intervention), and within 72 h after the 2nd intervention. The incidence of CIN was 2% after the 1st intervention (i.e., primary angioplasty) and 8% after the 2nd intervention. Patients with significant left ventricular systolic dysfunction after the 1st intervention (ejection fraction (EF) ≤35%) exhibited higher relative rises in creatinine levels after the 2nd intervention (18 ± 29% vs. 2 ± 16% for EF ≤35% and >35%, respectively, *p* = 0.03), while respective creatinine changes after the 1st revascularization procedure were comparable (−1 ± 14% vs. 2 ± 13%, *p* = 0.4). CIN after the 2nd intervention was over five-fold more frequent in subjects with low EF (28% vs. 5%, *p* = 0.007). The association between low EF and CIN incidence or relative creatinine changes after the 2nd intervention was maintained upon adjustment for baseline renal function, major CV risk factors, and the use of renin-angiotensin axis antagonists prior to admission. In conclusion, low EF predisposes to CIN after second contrast exposure in patients undergoing two-stage coronary angioplasty during the initial hospitalization for AMI. Our findings suggest a need of extended preventive measures against CIN or even postponement of second coronary intervention in patients with significant left ventricular dysfunction scheduled for the second step of staged angioplasty.

## 1. Introduction

According to current clinical practice guidelines, complete coronary revascularization should be considered before discharge in patients with acute myocardial infarction (AMI) and multivessel coronary artery disease (CAD) [1,2]. Most evidence supports benefits of multivessel angioplasty (as either a one-time or staged intervention) compared to culprit lesion-only intervention, which is primarily linked to a decreased need of urgent revascularization but also to beneficial trends in other clinical endpoints [2,3,4,5,6]. Multivessel angioplasty can be performed either during a single procedural session or as a two-stage intervention with the second step during the same hospitalization or after discharge.

There are no firm recommendations on the optimal timing of non-culprit lesion angioplasty or inter-procedural intervals in staged interventions [2]. The choice between these alternative approaches remains at the operator’s discretion and depends on multiple periprocedural and non-procedural issues affecting the net balance between individual clinical benefits and risks for each of the alternative management strategies.

Some meta-analyses [7] and large studies [8] suggest advantages of two-stage multivessel revascularization compared to immediate complex angioplasty in AMI, although contrary findings have also been reported [5,9]. Contrast load, usually higher at complex multivessel interventions, is one of main determinants of developing contrast-induced nephropathy (CIN) [10,11,12]. Importantly, patients who suffered post-procedural CIN are at an increased short-term and long-term risk of adverse cardiovascular events [11,12]. Thus, early identification of AMI subjects at high risk of CIN is necessary to optimize preventive measures in order to reduce CIN incidence and improve outcome.

To the best of our knowledge, risk factors for CIN after the 1st and 2nd interventions have not been estimated separately in AMI patients undergoing two-stage complex revascularization during the initial hospital stay. Our aim was to search for patient characteristics associated with changes in serum creatinine and CIN after each procedural session in AMI patients treated with staged multivessel coronary angioplasty.

## 2. Materials and Methods

### 2.1. Patients

We retrospectively pre-screened medical records of 138 patients (mean age 67 ± 11 years; 50 women and 88 men) with a final diagnosis of ST-segment elevation myocardial infarction (STEMI; 46%) or non-ST-segment elevation myocardial infarction (NSTEMI; 54%) and multivessel CAD on angiography, in whom complete angioplasty was performed as a staged procedure from December 2017 to October 2019 during the initial hospital stay in one of two centers of invasive cardiology. Exclusion criteria included: age below 18 years, hemodynamic instability (Killip class 3 or 4), significant left main coronary artery stenosis, mechanical complications of myocardial infarction, current chemotherapy, severe renal insufficiency (estimated glomerular filtration rate (eGFR) <30 mL/min per 1.73 m^2^), or renal-replacement therapy. The inter-procedural interval ranged from two to five days (median, three days). Iodixanol (Visipaque™, GE Healthcare, Chicago, IL, USA), an iso-osmolar contrast medium, was used in all procedural sessions. As standard CIN prophylaxis, isotonic sodium chloride was administered intravenously after each intervention and before the 2nd intervention with hydration regimens tailored to the patient’s clinical status [2]. No other pharmacologic strategies of CIN prevention were used in any of the study subjects.

The ethics committee of our University approved the study, including the fact that informed consent was not sought due to a retrospective study design (approval No.: 1072.6120.256.2019 issued on 21 November 2019).

### 2.2. Data Collection and Additional Calculations

Demographical, clinical, and biochemical patient characteristics were recorded from in-hospital medical records and discharge letters, including left ventricular ejection fraction (EF) assessed by echocardiography performed 0‒2 days after the 1st intervention (i.e., primary angioplasty) and validated by a senior echocardiographer. We recorded serum creatinine levels before the 1st intervention (at admission), within 72 h after the 1st intervention (before the 2nd intervention), and within 72 h after the 2nd intervention. CIN was defined by the widely used traditional criteria [13] as rises in circulating creatinine levels of ≥44 µmol/L or ≥25% from the baseline value, occurring within 72 h after intravascular contrast-medium administration in the absence of likely alternative explanations for kidney injury. The Chronic Kidney Disease Epidemiology Collaboration (CDK-EPI) formula was used to calculate baseline eGFR from serum creatinine on admission, age, and sex [14].

### 2.3. Statistical Analysis

Data are shown as mean ± standard deviation (SD) or proportions (%). As some data on serum creatinine were missing, 130 and 121 study subjects entered the final analysis with regard to post-procedural creatinine changes after the 1st and 2nd interventions, respectively (Table S1). Intergroup comparisons were performed by 2-tailed Fisher test and Student’s *t*-test (or Welch test in case of variance inhomogeneity by Levene’s test) for categorical and continuous variables, respectively. Correlates of post-procedural relative changes in serum creatinine after the 1st and 2nd interventions were estimated by means of Pearson’s or Spearman’s correlation coefficients (r). As preliminary data analysis identified an EF ≤35% as a risk factor for CIN after the 2nd but not 1st intervention, we also compared serum creatinine and its post-procedural changes for each angioplasty step according to the EF cutoff value of 35%. Multiple logistic regression or linear regression were used to identify independent determinants of CIN incidence or relative post-procedural creatinine changes, respectively, including age, gender, diabetes status, arterial hypertension, baseline eGFR and hemoglobin, EF after the 1st intervention, and the use of renin-angiotensin axis blockers prior to admission [15,16]. A *p*-value below 0.05 was considered as significant. Statistical analysis was performed using Statistica 13.3 (Statsoft Inc., Tulsa, OK, USA).

## 3. Results

After the 1st intervention (i.e., primary angioplasty) CIN was diagnosed in 3 of the 130 patients, none of whom developed CIN after the 2nd procedure. CIN occurred in 10 of the 121 subjects after the 2nd intervention (8%).

Owing to a low number of CIN episodes after the 1st intervention, intergroup comparisons were performed only according to the occurrence of CIN after the 2nd intervention. In patients who developed CIN after the 2nd intervention, the proportion of subjects with an EF ≤35% was significantly higher compared to their counterparts without this complication (50% vs. 12%, *p* = 0.007; Table 1). There were no intergroup differences in other patient characteristics (Table 1).

Relative creatinine changes after the 2nd intervention correlated inversely with EF as a continuous variable (*r* = −0.20, *p* = 0.03) and positively with low-density lipoprotein (LDL) cholesterol (*r* = 0.21, *p* = 0.03), whereas no associations with the remaining variables were found.

The incidence of CIN in subjects with an EF ≤35% was over five-fold increased after the 2nd intervention (28% vs. 5%, *p* = 0.007), but low and comparable after the 1st intervention (0% vs. 3%, *p* = 1) in comparison to those with an EF >35% (Table 2). Likewise, in the patients with low EF, post-procedural changes in serum creatinine were significantly higher after the 2nd intervention (18 ± 29 vs. 2 ± 16%, *p* = 0.03), but not after the 1st angioplasty (−1 ± 14% vs. 2 ± 13%, *p* = 0.4; Table 2).

Multivariate logistic regression identified an EF ≤35% as the only independent determinant of CIN occurrence after the 2nd angioplasty, with an odds of developing CIN elevated by over 10-fold in patients with an EF ≤35% compared to the remainder (odds ratio (OR): 13.8 (95% confidence interval: 1.5–125.7), *p* = 0.02; *p* = 0.7 by the goodness-of-fit Hosmer–Lemeshow test; Table 3). Likewise, on multiple regression, low EF independently predicted higher relative post-procedural creatinine changes after the 2nd intervention (non-standardized regression coefficient: 19 ± 6, *p* = 0.001), i.e., average relative creatinine changes were increased by 19% in those with EF ≤35% in comparison to EF >35%.

In contrast, this association was not observed with EF as a continuous variable (OR: 1.3, 95% confidence interval 0.9–2.0, per each EF decrement by 5%, *p* = 0.15; *p* = 0.3 by the Hosmer–Lemeshow test; Table 4), which suggests that the relationship between CIN and low EF was dependent mainly on higher CIN incidence in those with EF ≤35%.

Relative creatinine changes after the 1st intervention correlated positively with baseline eGFR (*r* = 0.26, *p* = 0.002). By multiple linear regression, increased relative changes of serum creatinine after the 1st intervention were independently associated with older age, hypertension and higher baseline eGFR, but not low EF (adjusted *R*^2^ = 0.21, *p* < 0.001; Table 5).

## 4. Discussion

The salient finding of our retrospective observational study is that an EF ≤35% turned out to be the key predictor of CIN after the 2nd intervention, but not the 1st intervention in AMI patients with multivessel CAD undergoing two-stage coronary angioplasty during the initial hospital stay. The fact that the association between low EF (≤35%) and CIN after the 2nd intervention was maintained after multivariate adjustment strengthens significant left ventricular (LV) systolic dysfunction as a predictor of CIN after the 2nd intervention.

### 4.1. Comparison with Other Studies—Incidence of CIN

In an observational study by Politi et al. [3], who compared outcomes between STEMI patients with multivessel CAD and without cardiogenic shock treated with culprit-only, immediate complex multivessel or two-stage angioplasty, the incidence of CIN, defined in the same way as in the present study, was 1.5‒3.6%. However, they did not report CIN incidence separately after the 1st and 2nd interventions in those undergoing two-stage in-hospital revascularization, and did not stratify their study subjects according to EF. Nevertheless, in that study EF averaged 45‒47%, similar to mean EF (49%) in our AMI patients with an EF >35%. When limiting the analysis to the latter subgroup, CIN incidence after the 2nd intervention in our patients (5%; Table 2) appears comparable with joint CIN incidence after two-stage multivessel intervention observed by Politi et al. (3.1%) [3].

Engstrøm et al. [4] observed rises over 50% in plasma creatinine in 2% of the participants of the Third DANish Study of Optimal Acute Treatment of Patients With STEMI: PRImary PCI in MULTIvessel Disease (DANAMI-3—PRIMULTI) trial, randomized into two-stage multivessel angioplasty with the second intervention performed after a median of two days from primary angioplasty for STEMI and multivessel CAD. Keeping in mind that median EF was about 50% in that trial, the results are also compatible with our study because the incidence of creatinine increases exceeding 50% was 1% and 2% after the 1st and 2nd interventions, respectively, in our study subjects without significant left ventricular dysfunction (mean EF: 49%).

### 4.2. Comparison with Other Studies—Low EF as a Predictor of CIN

Our results are in agreement with a comprehensive meta-analysis by He et al. [16] who described over five-fold higher odds of developing CIN for STEMI patients with an EF below 40%, i.e., close to the cut-off value defining low EF in the present study. This is also consistent with a study by Mehran et al. [17] who identified advanced congestive heart failure as an independent component of a risk score for prediction of CIN after percutaneous coronary intervention in 8357 patients. Moreover, depressed EF (≤40–45%) or congestive heart failure were present in 8 of the 12 validated prediction models for CIN after interventional cardiac procedures [15].

### 4.3. Mechanistic Considerations

CIN is linked to contrast-induced renal vasoconstriction, owing to cytotoxic endothelial and tubular injury and an imbalance between local vasoconstrictory mediators (endothelin and adenosine via type 1 adenosine receptors) and impaired nitric oxide bioavailability (in part due to reactive oxygen species formation), with a consequential fall in outer medullary blood flow, slower tubular and urinary flow rates, and medullary ischemia/reperfusion injury [11,12,18]. In addition, GFR decreases due to the activation of the tubuloglomerular feedback mechanism [11]. Since these abnormalities are also present in patients with low cardiac output exhibiting depressed renal perfusion, it does not seem implausible to assume that impaired renal blood flow, presumably accompanying low EF in STEMI patients, might account for an elevated incidence of CIN after the 2nd intervention in our subjects.

An analogous mechanism, i.e., potentiated susceptibility of the under-perfused kidney to contrast-induced acute kidney injury, either due to second contrast exposure or a higher contrast load during complex intervention at the time of primary angioplasty, might also be responsible for the different incidence of CIN between DANAMI-3—PRIMULTI [4], Complete versus Lesion-only PRImary PCI Trial (CvLPRIT) [5] and (CULPRIT Lesion Only PCI Versus Multivessel PCI in Cardiogenic SHOCK (CULPRIT-SHOCK) [19] trials according to EF. In particular, when comparing complex and culprit-only angioplasty, the incidence of the need of in-hospital renal-replacement therapy (representing severe CIN) was raised by about 50% (16% vs. 11%, respectively) in CULPRIT-SHOCK participants with AMI and cardiogenic shock (mean EF: 32%) [19]. In contrast, CIN incidence was the same for multivessel (1.4%) and culprit-only angioplasty (1.4%) in the CvLPRIT trial [5] recruiting STEMI subjects without cardiogenic shock at admission (mean EF: 45%). Notably, median contrast load was identical in the CvLPRIT [5] and CULPRIT-SHOCK [19] trials, averaging 250 mL and 190 mL for complex and culprit-only angioplasty, respectively.

Thus, the inter-trial differences in CIN risk after complex angioplasty could be mediated not by peri-procedural factors, but rather distinct patient characteristics, especially low cardiac output in the CULPRIT-SHOCK study. Additionally, in the DANAMI-3—PRIMULTI trial (mean EF: 50%), over 50% increase of serum creatinine occurred in a similar proportion of STEMI subjects with the culprit-only revascularization (2%) and those with the two-stage complex angioplasty performed after a median of two days from primary angioplasty of the infarct-related artery (1%) [4]. Finally, the proposed concept appears in agreement with no significant differences in CIN incidence between culprit-only (3.6%) and immediate (1.5%) or two-stage (3.1%) complex multivessel angioplasty in STEMI subjects studied by Politi et al. [3], with a mean EF of 45–47%, i.e., above the cut-off limit of 35%.

Therefore, it may be hypothesized that patients with low EF and consequent impairment of renal blood flow might be prone to contrast-induced subclinical kidney damage after the 1st intervention, even if mean serum creatinine in that subgroup virtually did not change and was similar to respective levels in the remainder, i.e., with EF >35%. However, due to insufficient kidney recovery during a median of three days before the 2nd intervention, the progress of discrete renal abnormalities to overt CIN could be triggered by an additional contrast load during next angioplasty.

For these reasons, intravenous hydration with isotonic saline, used as a routine inter-procedural CIN prophylaxis in our center, might have contributed to low CIN incidence after 1st intervention irrespective of EF, being yet less effective in CIN prevention after consecutive contrast administration to patients with low EF.

### 4.4. Clinical Implications

Our findings suggest that the risk of CIN after the second step of multivessel angioplasty is several-fold elevated in AMI patients with significant LV dysfunction even in the absence of relevant rises in serum creatinine after the 1st angioplasty. Therefore, as patients with considerably depressed EF appear particularly susceptible to CIN after the 2nd intervention, extended inter-procedural preventive measures are mandatory for CIN prophylaxis.

Optimal hydration with isotonic saline is an established prophylactic strategy with multiple benefits including increased renal perfusion and tubular flow rate, dilution of the contrast-medium concentration and lower intratubular viscosity, as well as inhibition of the renin-angiotensin axis and suppressed vasopressin release [2,10,11,12,18]. Moreover, on the basis of the Prevention of Contrast Renal Injury with Different Hydration Strategies (POSEIDON) trial which demonstrated a reduced risk of CIN by almost 60% in coronary patients with stable renal insufficiency, randomized to LV end-diastolic pressure (LVEDP)-guided intraprocedural and postprocedural hydration [20], a close monitoring of the hemodynamic status can be advisable to optimize the balance between appropriate renal perfusion and the risk of pulmonary congestion in AMI subjects with low EF. Notably, although Lima et al. [21] did not report any association between intraprocedural LVEDP and CIN incidence, Liu et al. [22] observed post-angioplasty CIN in as many as 36.5% of patients with both low EF (≤40%) and high LVEDP (≥20 mmHg), thereby being over four-fold more frequent compared to their counterparts with low EF but LVEDP < 20 mmHg.

In summary, beyond the above mentioned strategy of CIN prevention, the postponement of the 2nd intervention to ensure recovery from possible subclinical renal damage appears a plausible approach in AMI patients with an increased risk of CIN, including those with low EF, all the more because there are no firm recommendations on detailed timing of the treatment of non-culprit coronary lesions in AMI [1,2]. Moreover, the extended inter-procedural interval can increase renal benefits from high-dose statins [11], administered early as a standard of care in AMI [1], and also recommended for CIN prevention by current guidelines on myocardial revascularization [2].

Notably, in AMI patients receiving concurrent nephrotoxic medications predisposing to CIN [10,12] (e.g., commonly used non-steroidal anti-inflammatory drugs) prior to admission, a delay before the 2nd intervention might enable their full elimination after withdrawal, thereby decreasing the risk of the drug-induced adverse effects on renal function. Finally, the avoidance of too intensive blockade of the renin-angiotensin axis (linked by some authors to higher incidence of CIN [11,23]) may be prudent before the completion of two-stage multivessel angioplasty.

### 4.5. Study Limitations

First, our report is limited by a retrospective single-center design and a relatively small size of the study group, which profoundly constrains any far-going conclusions regarding determinants of CIN incidence. In addition, detailed procedural characteristics were unavailable in all the study subjects, which limited the set of covariates considered as potential determinants of CIN. However, we analyzed consecutive AMI patients with multivessel CAD treated with two-stage angioplasty. Moreover, uniform exclusion criteria were applied to decrease the heterogeneity of the study group. Additionally, in all our patients, the same contrast-medium was used and the only pharmacological strategy of CIN prevention was intravenous administration of isotonic saline according to similar hydration regimens. Thus, our findings, albeit preliminary, appear applicable to the vast majority of AMI subjects free of cardiogenic shock or severe renal insufficiency at admission.

Second, the inter-procedural interval varied from two to five days, which could also affect the results. However, the variability of this period was low, and in the majority of the study subjects the 2nd intervention was performed on the 3rd or 4th day after the 1st angioplasty.

Third, EF data after 1st intervention only was extracted from medical records, nonetheless, in agreement with current clinical practice guidelines [1], EF is not routinely measured at presentation in our AMI patients without hemodynamic instability or suspected mechanical complications in order to not delay emergency angiography and reperfusion treatment.

## 5. Conclusions

Low EF predisposes to CIN after second contrast exposure in patients undergoing two-stage coronary angioplasty during the initial hospitalization for AMI. Our findings may suggest a need of extended preventive measures against CIN or even postponement of the second coronary intervention in patients with significant left ventricular dysfunction scheduled for the second step of staged angioplasty. Nevertheless, prospective studies are warranted to unequivocally confirm our preliminary findings.

## Figures and Tables

**Table 1:** Characteristics of patients with and without contrast-induced nephropathy (CIN) after the second step of staged complete coronary revascularization during hospitalization for acute myocardial infarction (AMI).

Characteristic	CIN after 2nd Intervention *n* = 10	No CIN after 2nd Intervention *n* = 111	*p*-Value
Age (years)	68 ± 13	68 ± 11	0.9
Men/Women (%)	50/50	65/35	0.5
Body-mass index (kg/m^2^)	29.7 ± 5.0	28.3 ± 5.5	0.4
Hypertension (%)	78	81	1
Diabetes mellitus (%)	40	26	0.5
Smoking habit (%)	30	30	1
Atrial fibrillation at admission (%)	10	13	1
Killip class 2 at admission	10	7	0.6
Angiographic CAD			
Culprit artery, LAD/LCx/RCA (%)	5/2/3	42/26/32	0.9
Bifurcation procedure (%)	10	13	1
Two-vessel CAD (%)	80	72	0.7
Three-vessel CAD (%)	20	28	0.7
Radial access route at 2nd intervention (%)	90	86	1
Hemoglobin at admission (g/dL)	13.8 ± 1.7	13.8 ± 2.0	0.9
LDL cholesterol at admission (mmol/L)	3.9 ± 1.0	3.3 ± 1.3	0.2
HDL cholesterol at admission (mmol/L)	1.2 ± 0.4	1.2 ± 0.3	0.6
eGFR at admission (mL/min per 1.73 m^2^)	84 ± 21	76 ± 22	0.3
Creatinine at admission (µmol/L)	76 ± 19	87 ± 27	0.2
Creatinine after 1st intervention (µmol/L)	78 ± 18	87 ± 25	0.3
Creatinine after 2nd intervention (µmol/L)	115 ± 35	87 ± 26	**0.002**
EF after 1st intervention (%)	42 ± 13	46 ± 10	0.2
EF ≤35% after 1st intervention (%)	50	12	**0.007**
Hemoglobin after 1st intervention (g/dL)	13.2 ± 2.7	13.7 ± 2.1	0.5
Hemoglobin after 2nd intervention (g/dL)	12.1 ± 1.9	12.7 ± 2.2	0.4
ACEI or ARB prior to admission (%)	56	44	0.5
In-hospital medication (%)			
Aspirin	100	100	1
Clopidogrel	60	47	0.5
Ticagrelor	40	53	0.5
Statin	100	96	1
ACEI or ARB	70	90	0.09
Beta-blocker	100	96	1
Insulin	10	12	1

Data are shown as mean ± standard deviation (SD) or proportions (%). *p*-Values below 0.05 were marked as bold. ACEI: angiotensin-converting enzyme inhibitors; AMI: acute myocardial infarction; ARB: angiotensin receptor blockers; CAD: coronary artery disease; EF: ejection fraction; eGFR: estimated glomerular filtration rate; HDL: high-density lipoprotein; LAD: left anterior descending artery or its diagonal branches; LCx: left circumflex artery or its marginal branches; LDL: low-density lipoprotein; RCA: right coronary artery.

**Table 2:** Serum creatinine, its post-procedural changes and CIN incidence after the 1st and 2nd interventions according to low EF after the 1st angioplasty (≤35%) in patients undergoing two-stage complete coronary revascularization during hospitalization for AMI.

Characteristic	EF ≤35% (Mean EF: 28 ± 7%)	EF >35% (Mean EF: 49 ± 7%)	*p*-Value
Creatinine at admission (µmol/L)	97 ± 33	85 ± 25	0.12
Creatinine after 1st intervention (µmol/L)	95 ± 32	84 ± 22	0.16
Creatinine after 2nd intervention (µmol/L)	111 ± 34	86 ± 25	**0.007**
Creatinine change after 1st intervention (%)	−1 ± 14	2 ± 13	0.4
Creatinine change after 2nd intervention (%)	18 ± 29	2 ± 16	**0.03**
CIN incidence after 1st intervention (%)	0	3	1
CIN incidence after 2nd intervention (%)	28	5	**0.007**

Data are shown as mean ± SD or proportions (%). *p*-Value below 0.05 were marked as bold. Abbreviations as in Table 1.

**Table 3:** Multivariate logistic regression analysis of the predictors of CIN after the 2nd intervention with EF after the 1st angioplasty as a dichotomous variable (EF ≤35% vs. EF >35%).

Predictor	Odds Ratio (95% Confidence Interval)	*p*-Value
Age, per 10-year increase	0.7 (0.2‒2.3)	0.6
Gender, men vs. women	0.4 (0.05‒4.2)	0.5
Hypertension	0.4 (0.03‒4.2)	0.4
Diabetes mellitus	5.4 (0.6‒52.3)	0.15
ACEI or ARB prior to admission	6.7 (0.6‒71.1)	0.1
eGFR, per decrement by 10 mL/min per 1.73 m^2^	0.8 (0.4‒1.5)	0.5
Hemoglobin, per 1-g/dL decrease	0.9 (0.5‒1.5)	0.6
EF after 1st intervention ≤ 35%	13.8 (1.5‒125.7)	**0.02**

*p*-values below 0.05 are marked as bold. Abbreviations as in Table 1.

**Table 4:** Multivariate logistic regression analysis of the predictors of CIN after the 2nd intervention with EF after the 1st angioplasty as a continuous variable.

Predictor	Odds Ratio (95% Confidence Interval)	*p*-Value
Age, per 10-year increase	0.8 (0.3‒2.4)	0.7
Gender, men vs. women	0.6 (0.07‒5.1)	0.6
Hypertension	0.4 (0.04‒3.5)	0.4
Diabetes mellitus	4.0 (0.5‒32.8)	0.2
ACEI or ARB prior to admission	5.3 (0.6‒44.2)	0.13
eGFR, per decrement by 10 mL/min per 1.73 m^2^	0.8 (0.4‒1.4)	0.4
Hemoglobin, per 1-g/dL decrease	0.9 (0.5‒1.5)	0.7
EF after 1st intervention, per decrement by 5%	1.3 (0.9‒2.0)	0.15

Abbreviations as in Table 1.

**Table 5:** Multiple linear regression analysis of the predictors associated with relative creatinine changes after the 1st intervention.

Predictor	Non-Standardized Regression Coefficient ± SEM	*p*-Value
Age, per 10-year increase	3.8 ± 1.5	**0.01**
Gender, men vs. women	−3.7 ± 3.1	0.2
Hypertension	8.4 ± 3.5	**0.02**
Diabetes mellitus	−5.9 ± 2.9	0.05
ACEI or ARB prior to admission	0.6 ± 2.8	0.8
eGFR, per decrement by 10 mL/min per 1.73 m^2^	−3.0 ± 0.7	**<0.001**
Hemoglobin, per 1-g/dL decrease	0.9 ± 0.8	0.3
EF after 1st intervention ≤ 35%	−1.1 ± 3.7	0.8

*p*-Value below 0.05 are marked as bold. SEM: standard error of the mean; other abbreviations as in Table 1.

## Supplementary Materials

The following is available online at https://www.mdpi.com/2077-0383/9/6/1812/s1, Table S1: a fully anonymized dataset in MS-Excel format with key data supporting our findings (“Table S1 Dataset_CIN.xlsx”).
